# Bilateral Kienböck disease; a literature review and case presentation

**DOI:** 10.1016/j.tcr.2025.101138

**Published:** 2025-02-13

**Authors:** Ahmadreza Afshar, Mohammad Javad Shariyate, Ali Tabrizi

**Affiliations:** Department of Orthopedics, Imam Khomeini Hospital, Urmia University of Medical Sciences, Urmia, Iran

**Keywords:** Avascular necrosis, Bilateral Kienböck disease, Lichtman stage, Lunate osteonecrosis, Lunatomalacia, Ulnar variance

## Abstract

In this case report and comprehensive literature review, we present the prevalence and clinical variability of bilateral Kienböck disease, focusing on its frequency, gender and age distribution, ulnar variance, and the synchronicity and stage congruence of symptoms bilaterally. A systematic review of the literature and a detailed case presentation revealed that bilateral Kienböck disease, accounting for 3 to 7.3% of cases, may be underdiagnosed due to its variable and often asynchronous presentations between the left and right wrists. Our findings highlight the need for thorough bilateral evaluations in patients with Kienböck disease to ensure a comprehensive diagnosis and to guide optimal management. This study advocates for heightened clinical vigilance and advances a nuanced understanding of this complex condition.

## Introduction

The avascular necrosis of the lunate, identified through radiographic changes by Robert Kienböck in 1910, is known as Kienböck disease [[1], [2], [3], [4]]. While traditionally observed as a unilateral condition predominantly affecting the dominant wrist, bilateral cases have been increasingly reported, expanding the understanding of its etiology and progression [[4], [5], [6], [7], [8], [9], [10], [11], [12], [13], [14], [15], [16], [17], [18], [19], [20], [21], [22], [23], [24], [25]]. Despite the research into the manifestation of Kienböck disease, the bilateral occurrence remains enigmatic due to limited study and understanding. Its pathogenesis is suggested to involve a combination of traumatic, mechanical, circulatory, and systemic factors, each contributing to the multifaceted nature of this wrist pathology.

Bilateral Kienböck disease, though infrequent, poses significant challenges in diagnosis and treatment due to its variable presentation and the potential for asynchronous symptom development. The inaugural report of bilateral Kienböck's disease by Wette in 1931, as cited by Steinhäuser and Posival, marked the beginning of its recognition in medical literature [23]. Since then, scholarly attention to bilateral Kienböck's disease has been sparse [[4], [5], [6], [7], [8], [9], [10], [11], [12], [13], [14], [15], [16], [17], [18], [19], [20], [21], [22], [23], [24], [25], [26]], leaving many aspects of its clinical presentation and progression underexplored.

This paper contributes to the existing body of knowledge by presenting a case of bilateral Kienböck disease and conducting a systematic literature review. Our goal is to delineate the characteristics of bilateral Kienböck disease, such as its frequency, average age of onset, sex distribution, and ulnar variance. We also aim to examine the synchronicity of symptom presentation and disease staging between the left and right wrists at the time of diagnosis. Through this investigation, we seek to deepen the understanding of bilateral Kienböck disease, highlight its challenges in diagnosis, and discuss the implications for clinical practice. This comprehensive review, enriched by our case study, endeavors to foster dialogue on the management and prognosis of this complex condition.

## Case report

A 48-year-old male, a professional mason, presented with bilateral wrist discomfort and swelling. The patient reported a history of surgical intervention involving fixation of a right radius fracture with plate and screws twenty years prior, with subsequent removal of the hardware two years after the surgery. The onset of symptoms in the left wrist occurred two years before presentation, whereas symptoms in the right wrist emerged one year prior.

On evaluation, the patient's pain intensity, assessed using the Visual Analog Scale (VAS) where 0 represents no pain and 10 represents the worst pain imaginable, was rated 10 for the left wrist and 5 for the right wrist. Objective assessment revealed a power grip strength of 9 kg in the left hand and 20 kg in the right hand. Pinch grip strength was measured at 8.5 kg for the left hand and 10 kg for the right hand. The range of motion was noted to be 40° flexion and 30° extension for the right wrist and 50° flexion and 30° extension for the left wrist. The supination and pronation rotation of the left forearm was within normal limits, whereas the right forearm exhibited restricted rotation, limited to 45° for both supination and pronation.

**Fig. 1:**
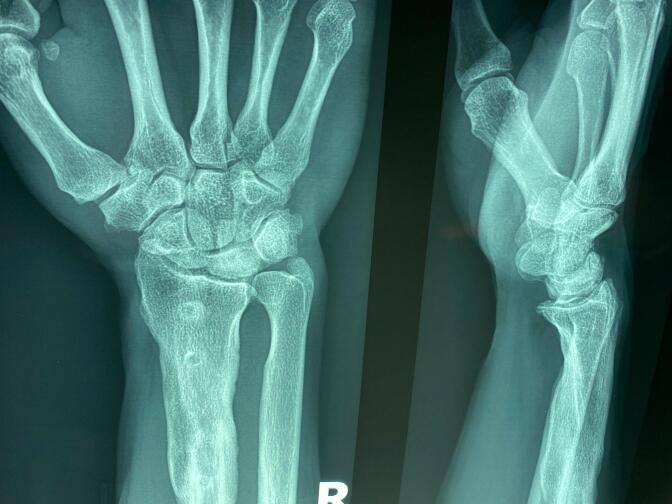
Antroposterior and lateral plain radiographs of the right wrist demonstrates increased density, sclerosis of the lunate and + 2 mm positive ulnar variance (Lichtman stage II).The effects of the previous radius fixation with plate and screws were obvious on the right radius.

**Fig. 2:**
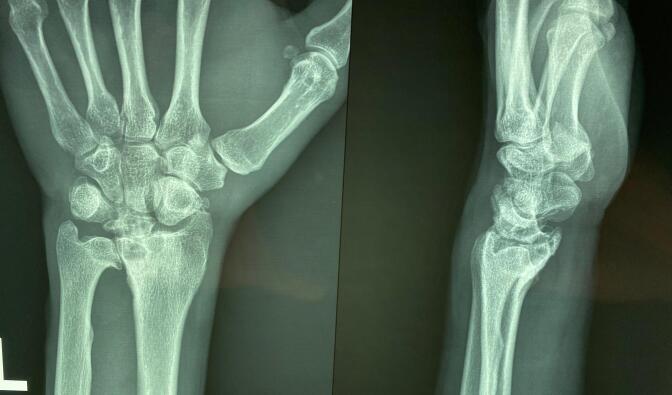
Antroposterior and lateral plain radiographs of the left wrist demonstrates collapse of the lunate, zero ulnar variance and advance degeneration of the radiocarpal and rdioulnar joints (Lichtman stage IV).

**Fig. 3:**
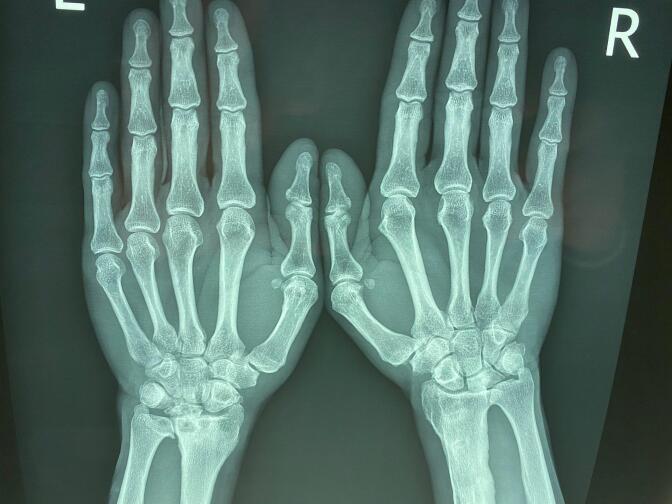
Antroposterior plain radiograph of the right and left wrist in one frame.

**Fig. 4:**
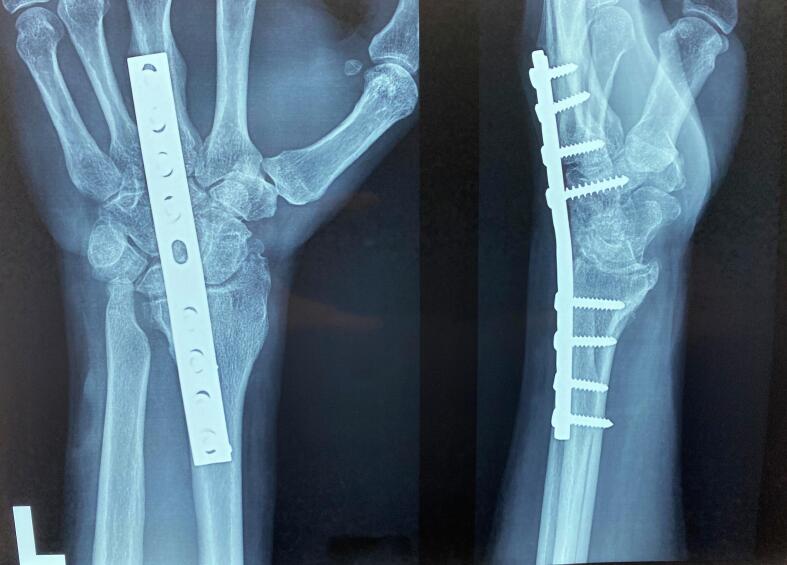
Antroposterior and lateral plain radiographs of the left wrist demonstrates six months post arthrodesis surgery with plate and screws. Bower's hemiresection-interposition arthroplasty was performed for distal radio-ulnar arthrosis.

Diagnostic imaging included plain radiographs of both wrists. The right wrist radiograph revealed increased density and sclerosis of the lunate, accompanied by a +2 mm positive ulnar variance. The radiograph also indicated the presence of sequelae from the previous radius fixation (Fig. 1). In contrast, the left wrist radiograph showed collapse of the lunate, zero ulnar variance, and advanced degeneration of the radiocarpal and radioulnar joints (Fig. 2). Based on the Lichtman classification for Kienböck's disease, the left wrist was determined to be at stage IV, and the right wrist was at stage II. Fig. 3 demonstrates antroposterior plain radiograph of the right and left wrist in one frame. Given the patient's reported severity of pain and the advanced stage of disease in the left wrist, wrist arthrodesis was offered and performed (Fig. 4).

## Discussion

A comprehensive literature search and review on bilateral Kienböck disease was conducted in January 2024, utilizing PubMed (www.pubmed.com), Google (www.google.com), Science Direct (http://www.sciencedirect.com), and Springer (http://link.springer.com) databases. The search strategy incorporated the keywords “bilateral Kienböck disease” and “bilateral aseptic necrosis of the lunate.” References and cross-references of all identified articles were meticulously examined for relevance and inclusion.

Osteonecrosis may be seen in different bones and major joints; however, Kienböck disease is described as an isolated osteonecrosis of the lunate. Therefore, we focused on isolated osteonecrosis of the lunate and excluded studies reporting concurrent aseptic necrosis of other bones. Articles published before the introduction of Lichtman's radiographic classification in 1977 were excluded, acknowledging the significance of this classification in the diagnostic and staging framework of Kienböck disease [27].

Steinhauser and Posival published the first article on bilateral Kienböck disease in 1982, in which they reported that 32 patients had been previously diagnosed with bilateral Kienböck disease [22]. Our literature review revealed a limited number of publications addressing bilateral Kienböck disease since 1982, with 22 publications documenting 50 cases. Publication language breakdown was English (18), French (1), Danish (1), German (1), and Russian (1). A limited number of articles failed to include the Lichtman stage and ulnar variance in their text, so we examined the presented pictures for the Lichtman stage and ulnar variance and marked our findings with * in Table 1, however, we were unable to identify Lichtman stage and ulnar variance of the patients in some. Kristensen et al. (1986), Rasmussen and Schantz (1987), and Viljakka et al. (1987) did not identify the characteristics of their cases [14,17,26]. Finally, data from 40 subjects from 19 articles [[5], [6], [7], [8], [9], [10], [11], [12], [13],^15^,^16^,[18], [19], [20], [21], [22], [23], [24], [25], [26]] and the data from the current case were pooled in Table 1 to demonstrate the characteristics of 40 bilateral Kienböck disease patients.

**Table 1:** Characteristics of the reported patients with bilateral Kienböck disease⁎

Author	Gender/Age	Lichtman stage		Ulnar variance (mm)		Time period between the both sides Symptoms onset (year)	Past medical history	Corticosteroid consumption	Occupation
		Right	Left	Right	Left				
Steinhäuser and Posival [23]	M/41	NI	NI	-2	-3	18 year			Brick layer
	M/28	NI	NI	-2	-2	1 year			Brick layer
	M/45	NI	NI	-2	-2	12 year			Terrazzo layer
	M/41	NI	NI	-1	-1	3 year			Turner
	M/49	NI	NI	-3	-3	7 year			Unskilled worker
	F/27	NI	NI			2 year			Dental technician
Morgan and McCue [16]	M/40	III	III	Minus	Minus	Concurrent			Railroad mechanic
	M/26	III	II	Minus	Minus	Concurrent			Shipyard labor
Kahn and Bade [13]	F/56	II	IV	Minus⁎	Minus⁎	Concurrent	Rheumatologic consultation		NI
Agus [5]	F/62	NI	NI	NI	NI	Concurrent	Systemic sclerosis		Housewife
Gelineck and Mikkelsen [8]	M/49	IV⁎	IV⁎	-4	-2	25 years			Truck driver
Blachier et al. [6]	F/14	IIIA	IIIA	-5	-5	Concurrent	Obesity		NI
Mok et al. [15]	M/19	IV	II	Minus⁎	NI	4 months	SLE		Student
Edmunds and Harvey [9]	F/62	III	III	0	0	Concurrent	Hemodialysis	+	NI
Taniguchi et al. [24]	M/21	III	III	0	0	Concurrent			Manual laborer
	F/43	IV	IV	0	0	Concurrent			Manual laborer
	F/34	I	II	+1	+2	Concurrent	SLE	+	Housewife
	F/65	III	II	+4	+3	Concurrent	Lupoid hepatitis	+	Retired
	F/62	IV	IV	+3	+5				Retired
Rennie et al. [18]	M/31	II	II	Minus	Minus	2 years	Scleroderma		Mechanist
Yazaki et al. [25]	M/28	IIIB	NI	NI	NI	21 years			Car mechanic
	M/51	IV	IIIB	NI	NI	NI			Plumber
	M/47	NI	IIIB	NI	NI	NI			Construction worker
	M/45	IIIB	II	NI	NI	NI			Driver/construction worker
	M/52	IIIA	IIIB	NI	NI	NI			Factory worker
	M/49	IIIA	IIIB	NI	NI	NI			Welder
	M/57	IIIB	IIIB	NI	NI				Tile maker
	M/33	IIIB	IIIB	NI	NI	15 years			Professional bass angler
	M/14	IIIA	IIIA	NI	NI	Concurrent			Baseball player
	M/44	IIIB	IIIB	NI	NI	Concurrent			Truck driver
	F/23	IIIA	IIIA	NI	NI	3 years			Care worker
Seah et al. [19]	F/25	IIIA	I	O⁎	O⁎	A few months	Type 1 diabetes		Student
Son et al. [22]	F/57	IIIB⁎	IIIB⁎	NI	NI	Concurrent	MCTD	+	NI
Shimizu et al. [21]	M/14	IIIA	IIIA	-5	Minus⁎	3 months			Basketball player
Goloborod'ko [11]	M/63	IIIB	IIIB	Minus⁎	Minus⁎	1 year			Janitor
Sekej and Rupreht [20]	M/28	IIIA	IIIA	-3	-2.5	Concurrent			NI
Farah et al. [10]	F/63	IIIB	IIIB						NI
Huang et al. [12]	M/14	IIIA	II	Minus	Minus	Concurrent	Factor V Leiden thrombophilia		NI
Chouhan et al. [7]	M/38	IIIB	IIIA	Minus⁎	Minus⁎	Concurrent	Gouty arthritis		NI
The current case	M/48	II	IV	+2	0	1 year			Mason

M: male; F: female; NI: not indicated;

⁎ Marks our findings about ulnar variance on the articles' pictures.

The demographic analysis of 40 bilateral cases highlighted a male predominance (male-to-female ratio of approximately 2:1; 27 male and 13 female) with a mean age of 40.2 years (SD ± 15, range 14-63 years). The distribution of ulnar variances was notably varied, with 33 cases demonstrating ulnar negative variance, nine cases with ulnar neutral, and seven cases with ulnar positive variance. However, for 31 wrists, the ulnar variance was not identified. This variation underscores the complexity of Kienböck disease and the potential role of ulnar variance in its pathogenesis.

Notable past medical history included 12 patients with systemic disorders and four patients with a history of corticosteroid use. 25 patients were occupationally involved in heavy manual work. Three out of the four 14-year-old (skeletally immature) patients were involved in heavy sports. Therefore, the occupations and activities of patients, particularly those involving heavy manual work or sports, align with known risk factors for the unilateral manifestation of the disease.

Examining the onset of bilateral symptoms, onset of symptoms between the right and left wrists was variable with approximately equal numbers of patients reporting simultaneous onset or a time lag. 16 patients had concurrent bilateral symptoms, while 16 patients had a time difference from a few months to 25 years. In eight patients, the time difference of the symptom onset was not mentioned. With the Lichtman stages, 13 patients exhibited distinct stages of Kienböck disease on the right and left sides, while 19 patients presented the same stage. Eight patients lacked sufficient information regarding their Lichtman stage.

The incidence of bilateral Kienböck disease has been estimated between 3 and 7. 3 % of all cases [14,17,[23], [24], [25], [26]]. In 1987, Viljakka et al. reported 4 (7. 3%) bilateral involvement out of 55 Kienböck disease patients [26], Kristensen et al. reported 3 (6. 5%) bilateral involvement out of 46 Kienböck disease patients and [14] Rasmussen et al. reported bilateral involvement occurred in three (3%) of 93 patients with Kienböck disease [17]. Taniguchi et al. reported that 5 (4 %) out of 126 Kienböck disease patients had bilateral involvement, which showed mean of +1.8 mm (0–5) ulnar variance. However, Taniguchi and Yazaki hold an opposing view. Taniguchi et al. found that negative ulnar variance was not a major risk factor in the five bilateral Kienböck disease patients [24]. Yazakiet et al. found that 11 (4 %) out of 251 Kienböck disease patients had bilateral involvement. Yazakiet al. could not identify any radiographic parameters that served as risk factors for bilateral Kienböck disease compared to unilateral Kienböck disease, including ulnar variance, in eleven patients [25]. Steinhäuserand and Posival reported that 6 (7%) out of 85 Kienböck disease patients had bilateral involvement. All the 6 cases had ulnar minus variants on both sides [23]. The variation in reported incidence rates reported in literature may reflect differences in study populations, diagnostic criteria, and the sensitivity of radiographic assessment. The above findings highlight the variability in incidence rates and the ongoing debate regarding the risk factors and pathogenesis of bilateral Kienböck disease.

Taken as a whole, our analysis suggests that bilateral Kienböck disease exhibits a similar clinical presentation to unilateral cases, with no distinct differences in demographic characteristics [[1], [2], [3]]. However, variability in symptom onset, combined with the observation that Lichtman stages can differ between wrists, underscores the heterogeneity of bilateral Kienböck disease and suggests a complex interplay of individual risk factors and disease progression mechanisms.

Furthermore, our review challenges the assumption that negative ulnar variance is a predominant risk factor in bilateral cases, echoing the findings of Taniguchi et al. and Yazaki et al., who reported no significant correlation between ulnar variance and bilateral disease occurrence.

Bilateral Kienböck disease, while rare, presents a significant diagnostic and therapeutic challenge. The variability in symptom onset, the potential for asymptomatic disease on one side, and the variability in Lichtman stages between wrists necessitate a vigilant approach to diagnosis and management. This review and case report highlight the importance of considering the possibility of bilateral involvement in patients presenting with unilateral Kienböck disease symptoms and the need for further research to elucidate the pathogenesis and optimal management strategies for this complex condition. The findings also imply that the incidence of bilateral Kienböck disease may be underreported, urging clinicians to maintain a high suspicion index, undertake comprehensive bilateral assessments, and follow up with patients affected by unilateral Bilateral Kienböck disease.

## CRediT authorship contribution statement

**Ahmadreza Afshar:** Conceptualization, Data curation, Methodology, Supervision, Writing – original draft, Writing – review & editing. **Mohammad Javad Shariyate:** Conceptualization, Data curation, Methodology, Writing – original draft, Writing – review & editing. **Ali Tabrizi:** Conceptualization, Methodology, Writing – original draft, Writing – review & editing.

## Informed consent

Written informed consent was obtained from the patient for patient's anonymized information to be published in this article.

## Ethical approval

The study was approved by the medical research and ethics committee of the Urmia University of Medical Sciences with registered Numbers: ir/IR.UMSU.REC.1402.325.

Available at: https://ethics.research.ac.ir/IR.UMSU.REC.1402.325

## Funding statement

The authors received no financial support for the research, authorship, and/or publication of this article.

## Declaration of competing interest

The authors declare that they have no known competing financial interests or personal relationships that could have appeared to influence the work reported in this paper.

## References

[1] Daly C.A., Graf A.R. (2022). Kienböck disease: clinical presentation, epidemiology, and historical perspective. Hand Clin..

[2] Lutsky K., Beredjiklian P.K. (2012). Kienböck disease. J. Hand. Surg. [Am.].

[3] Beredjiklian P.K. (2009). Kienböck’s disease. J. Hand. Surg. [Am.].

[4] Lichtman D.M., Pientka W.F., Bain G.I. (2016). Kienböck disease: moving forward. J. Hand. Surg. [Am.].

[5] Agus B. (1987). Bilateral aseptic necrosis of the lunate in systemic sclerosis. Clin. Exp. Rheumatol..

[6] Blachier D., Renaux P., Forestier A., Mourad G. (1992). Maladie de Kienböck bilatérale chez l’adolescent. A propos d’un cas traité chirurgicalement [Bilateral Kienbock’s disease in adolescents. Apropos of a surgically treated case]. Rev. Chir. Orthop. Reparatrice Appar. Mot..

[7] Chouhan D., Shankar V., Ansari M.T. (2020). Bilateral Kienböck’s disease concomitant with gouty arthritis. BMJ Case Rep..

[8] Gelineck J., Mikkelsen S.S. (1987). Bilateral morbus Kienböck [Bilateral Kienböck’s disease]. Ugeskr. Laeger.

[9] Edmunds I., Harvey F.J. (1998). Bilateral Kienbock’s disease in a patient on haemodialysis. Aust. N. Z. J. Surg..

[10] Farah F., Kalaydjian A., Miller J., Scemama de Gialluly P. (2019). SCS therapy in a patient with advanced bilateral Kienbocks. Pract. Pain Manag..

[11] Goloborod’ko S.A. (2016). Bilateral Kienböck’s disease in an elderly patient. Novosti Khirurgii..

[12] Huang T.C., Li J., Moran S.L. (2019). Bilateral Kienböck’s disease in a child with Factor V Leiden thrombophilia: a case report. J. Hand Surg. Eur..

[13] Kahn M.L., Bade H.A. (1986). Lunate osteomyelitis in a patient with bilateral Kienböck’s disease. Orthop. Rev..

[14] Kristensen S.S., Thomassen E., Christensen F. (1986). Kienböck’s disease—late results by non-surgical treatment. A follow-up study. J. Hand Surg. (Br.).

[15] Mok C.C., Lau C.S., Cheng P.W. (1997). Bilateral Kienbock’s disease in SLE. Scand. J. Rheumatol..

[16] Morgan R.F., McCue F.C. (1983). Bilateral Kienböck’s disease. J. Hand. Surg. [Am.].

[17] Rasmussen F., Schantz K. (Aug 1987). Radiologic aspects of lunatomalacia. Eur. J. Radiol..

[18] Rennie C., Britton J., Prouse P. (1999). Bilateral avascular necrosis of the lunate in a patient with severe raynaud’s phenomenon and scleroderma. J. Clin. Rheumatol..

[19] Seah K.T., McEachan J., Davidson D. (2012). Bilateral Kienbock’s disease in association with type 1 diabetes. J. Hand Surg. Eur..

[20] Sekej M., Rupreht M. (2017). Bilateral Kienböck’s disease with bilateral negative ulnar variance: a case report. Acta Medico-Biotechnica..

[21] Shimizu T., Yajima H., Omokawa S., Murata K., Kobata Y., Kawamura K., Tanaka Y. (2013). Bilateral pediatric Kienböck’s disease: a case report. J. Pediatr. Orthop. B.

[22] Son C.N., Lee S., Joo K.B., Jun J.B. (2013). An unusual cause of wrist pain; Kienbock’s disease. J. Rheum. Dis..

[23] Steinhäuser J., Posival H. (1982). Doppelseitige Mondbeinnekrose. Ein Beitrag zur Pathogenese [Bilateral necrosis of the lunate bone. A pathogenic study]. Z. Orthop. Ihre Grenzgeb..

[24] Taniguchi Y., Tamaki T. (1998). Bilateral Kienböck’s disease. J. Orthop. Sci..

[25] Yazaki N., Nakamura R., Nakao E., Iwata Y., Tatebe M., Hattori T. (2005). Bilateral Kienbock’s disease. J. Hand Surg. (Br.).

[26] Viljakka T., Vastamäki M., Solonen K.A., Tallroth K. (1987). Silicone implant arthroplasty in Kienböck’s disease. Acta Orthop. Scand..

[27] Lichtman D.M., Mack G.R., MacDonald R.I., Gunther S.F., Wilson J.N. (1977). Kienböck’s disease: the role of silicone replacement arthroplasty. J. Bone Joint Surg. Am..

